# Pathways to meaning: how family functioning shapes meaning in life through school belonging among Chinese adolescents

**DOI:** 10.3389/fpsyg.2026.1889582

**Published:** 2026-08-27

**Authors:** Xiaoxia Deng, Jie Cao, Jinhua Li, Beni Widarman Yus Kelana

**Affiliations:** 1 School of Educational Science, Hunan University of Science and Engineering, Yongzhou, Hunan, China; 2 Faculty of Education, Xiangnan University, Chenzhou, Hunan, China; 3 Faculty of Arts and Design, Xiangnan University, Chenzhou, Hunan, China; 4 Azman Hashim International Business School, Universiti Teknologi Malaysia, Johor Bahru, Johor, Malaysia

**Keywords:** adolescents, China, family functioning, meaning in life, school belonging

## Abstract

**Introduction:**

The relationship between family functioning, school belonging, and meaning in life has been rarely investigated in China, especially among early adolescents. Drawing on the Overlapping Spheres of Influence (OSI) theory, this study aims to explore the impact of family functioning on adolescents’ meaning in life and to examine the mediating role of school belonging in this relationship.

**Methods:**

A multi-stage stratified sampling method was employed to recruit 410 valid participants from middle schools in China. Data were analyzed using Partial Least Squares Structural Equation Modeling (PLS-SEM) to test both the measurement and structural models, including mediation analysis via bootstrapping with 5,000 resamples.

**Results:**

The results indicated that both family functioning and school belonging positively predict meaning in life among adolescents. Furthermore, school belonging can mediate the relationship between family functioning and meaning in life, accounting for 55.6% of the total effect. The model explained 33.0% of the variance in meaning in life, with adequate predictive relevance.

**Conclusions:**

These findings confirm that family functioning influences adolescents’ meaning in life both directly and indirectly through school belonging, with the indirect pathway being stronger. This highlights that a supportive family environment fosters psychological readiness for adolescents to connect with school, where their meaning in life is largely constructed. The study also offers practical implications for educators, mental health professionals, and policymakers to promote family-school partnerships and strengths-based interventions that enhance adolescent well-being.

## 1 Introduction

Adolescence is a critical developmental stage characterized not only by rapid physical and cognitive changes but also by the profound formulation of identity. Along with cognitive development, children begin to construct a complex sense of self. As early as James (1890), scholars have recognized that the self comprises different dimensions, including the material self, the social self, and the spiritual self, which encompasses beliefs, values, and internal states (Harter, 1988, 2012). Thus, the pursuit of meaning in life, as a core aspect of the spiritual self and a reflection of who one is, is inherent in human nature (Erikson and Erikson, 1998). For adolescents, developing a coherent sense of meaning is a fundamental psychological need that buffers against psychological distress and promotes overall wellbeing.

However, the modern adolescent journey is often fraught with challenges, and some teenagers are prone to emotional or behavioral problems during this process. The Report on the Development of National Mental Health in China (2023–2024) indicates that the prevalence of emotional and behavioral difficulties affects approximately 30 million Chinese adolescents under the age of 17 (Sun and Jiang, 2025). At the same time, it is noted that between 15 and 20% of Chinese junior high school pupils are diagnosed with depression. These all underscore the pervasive impact of these conditions on adolescent wellbeing (Chen et al., 2021). To address this public health challenge, researchers have explored protective factors that mitigate psychological distress. Among these, meaning in life has emerged as an important buffer, with empirical evidence linking it to enhanced mental health resilience (Chen et al., 2021; Steger et al., 2015). Adolescents who report a robust meaning in life demonstrate significantly positive emotional wellbeing and physiological resilience (Lin et al., 2021). While deficiencies in meaning in life are strongly associated with adverse mental health outcomes, including increased vulnerability to self-harming behaviors (Hu et al., 2023).

As a core psychological construct, meaning in life is considered as an individual’s understanding of their own existence in terms of its purpose, value, and consistency (Steger et al., 2006). In the field of adolescent development, the sense of meaning of life is not only regarded as a key indicator of psychological wellbeing but also plays an important protective role. Studies displayed that adolescent with a strong sense of life is linked to reduced depression and suicidal ideation, which are significantly related to higher life satisfaction, psychological resilience and positive academic engagement (Klutsey and Mahama, 2025; Lin et al., 2025; Onyekachi et al., 2024; Zhou et al., 2025). Therefore, promoting the growth of adolescents’ meaning in life is considered the cornerstone of active adolescent development and preventive mental health intervention, which is highly consistent with the core mission of school mental health (Yang et al., 2023).

The family serves as the primary and most enduring environment where this early sense of self and meaning is cultivated. For adolescents, a high-functioning family acts as a secure emotional base, providing the necessary psychological safety and structural support to internalize a positive sense of meaning in life (Chen et al., 2023). However, family environments are not uniformly beneficial. Some families are dysfunctional and can have profound negative effects on child development (Jacome-Mora et al., 2025; Lamborn et al., 1991). Research consistently shows that dysfunctional family dynamics, often marked by heightened tensions and disputes, can result in internalized difficulties like anxiety or externalized behaviors (Soloski et al., 2016). Similarly, families characterized by less warmth and unsupportive parenting styles have been related to a plethora of maladjustment outcomes in children and adolescents (Alcaide et al., 2025; Darling and Steinberg, 1993; Jacome-Mora et al., 2025). Therefore, the quality of family functioning, whether it provides nurturing scaffolding or introduces developmental risks, is a critical determinant of an adolescent’s psychological trajectory.

As adolescents transition into wider social spheres, the school environment becomes a secondary, yet equally vital, developmental field. Entering junior high school, teenagers’ cognitive ability is experiencing a leap in the stage of formal operation in Piaget’s theory, which enables them to systematically reflect on abstract propositions such as life, death, and value for the first time, thus laying a cognitive foundation for consciously pursuing meaning (Oesterdiekhoff, 2024; Piaget, 2016). During this period, the source of meaning in life is no longer limited to familial values but is increasingly shaped by the sense of belonging acquired at school. Previous studies indicated that school belonging can significantly affect the meaning in life among students (Ramirez et al., 2021; Yıldırım et al., 2023; Zhang et al., 2018). Given that emerging adults dedicate substantial time to schools, their social embeddedness within academic communities may further cultivate life purpose. The Overlapping Spheres of Influence (OSI) theory further emphasizes the interconnecting role of both schools and families to students’ development, highlighting that these two environments jointly shape a student’s existential worldview.

Despite these foundational findings, several critical gaps in knowledge remain. First, while the impacts of family functioning and school belonging on mental health have been explored independently, the internal mechanism through which family dynamics translate into an internalized sense of meaning in life remains under-investigated, particularly within the developmental context of Chinese early adolescence. Second, although the OSI theory suggests that schools and families function as interconnected spheres, there is limited empirical evidence clarifying whether school belonging serves as a necessary mediating bridge that extends the protective effects of a nurturing family environment to an individual’s existential framework. Third, most studies on meaning in life have focused on university students or adults, leaving a gap in understanding how these ecological systems interact during the unique developmental window of junior high school, where identity formation and the search for meaning are most volatile.

Thus, this study first detects the influence of family functioning and school belonging on students’ meaning in life. It then examines the mediating effect of school belonging. Through this work, the research enriches existing literature through various contributions. Theoretically, this study is helpful to deepen the understanding of OSI theory. As a microscopic system of individual growth, family functioning may further shape the adaptability of middle school students to the school environment by influencing their sense of security and self-identity. As an intermediary variable, school belonging may extend the effectiveness of family support to a wider social situation and form an individual’s cognitive framework for the meaning of life. The study of this interaction mechanism can provide empirical support for the OSI theory, and at the same time reveal the dynamic influence path of different environmental levels on adolescent psychological development. In the practical part, this study can provide scientific basis for educational practice. At present, adolescents are faced with multiple dilemmas such as academic pressure, social challenges and identity confusion, and some students are deficient in a sense of meaning in life and may even have mental health troubles (Suo et al., 2024). If the synergy between family functioning and school belonging can be verified to promote meaning of life, educators can design more targeted intervention programs.

## 2 Theoretical foundation and hypotheses development

### 2.1 The theory of overlapping spheres of influence (OSI)

Based on the social capital theory and ecosystem theory, Epstein et al. (2002) put forward the OSI theory with caring as the core and constructed a new theoretical paradigm and practical mechanism of family, school, and community. The OSI proposes that a child’s development and learning thrive most effectively when family, school, and community function as interconnected, overlapping spheres. Each sphere serves as a critical environment shaping a child’s social and academic progress, with their combined interactions forming a dynamic support network. The degree of overlap between these spheres may shift as a child matures or circumstances evolve, influencing how seamlessly these settings collaborate to nurture educational outcomes. Proactive communication and cooperation among these spheres cultivate a unified support framework, bolstering both academic performance and holistic wellbeing. The OSI theory includes external model and internal model, and puts forward six modes of family, school, and community participation, namely, parenting, communication, volunteer service, home study, decision-making and community cooperation (Epstein et al., 2002).

### 2.2 The meaning in life

The concept of meaning in life refers to people’s understanding of their purpose and inherent worth, which reflects the individual’s pursuit of value and goal and his perception of whether his life is meaningful (Steger et al., 2006). It embodies a relativistic perspective in which individuals create their own meanings based on their personal experiences, values, and goals. When individuals gain clarity about the meaning of their existence, they are naturally driven to pursue goals that align with this understanding. Conversely, if one fails to discern the purpose of their being, they may succumb to a profound sense of confusion and emptiness, ultimately losing hope and direction in life (Zhang J. et al., 2024; Zhang Z. et al., 2024). Teenagers with high meaning of life can show stronger positive emotions and physical health (Lin et al., 2021). On the contrary, an absence of meaning in life has been empirically linked to adverse psychological outcomes, including poor mental health and self-injury (Chen et al., 2021; Hu et al., 2023).

### 2.3 Family functioning

Family is essential to the development of all family members. Family functioning is defined as individual’s subjective perception and satisfaction evaluation of family support quality (Smilkstein, 1978). It is commonly conceptualized as a family system’s ability to effectively maintain emotional cohesion, establish adaptive relational patterns, facilitate intermember communication, and collectively respond to psychosocial stressors (Olson et al., 1983; Peng et al., 2023). Family functioning covers five core dimensions: the degree to which individuals get help from their families when facing difficulties (adaption), the degree to which family members discuss affairs together and participate in decision-making (partnership), the degree to which families support individual’s independent growth and development (growth), emotional interaction and intimate experience among family members (affection), and individual’s satisfaction with sharing family time and resources (resolve) (Smilkstein, 1978). Family functioning is a more robust predictor of adolescent psychological wellbeing than objective family structures (Chen et al., 2023). While parenting styles often focus on unidirectional influence, family functioning encompasses the reciprocal emotional support that is particularly salient during early adolescence (Peng et al., 2023).

Furthermore, a key indicator of this mutual support is the quality of ties between children and their parents. Recent empirical research has underscored that positive parent–child dynamics, marked by parental warmth, secure attachment, and constructive communication, are essential for a healthy family system and serve as a strong shield against mental health challenges (He et al., 2025; Zhu et al., 2023). Particularly in the Chinese context, positive child–parent interactions have been consistently identified as powerful protective factors that enhance adolescents’ self-esteem, psychological wellbeing, and ultimately foster a stronger sense of meaning in life (Shek et al., 2021; Tian et al., 2013). For adolescents, a high-functioning family with supportive parent–child ties acts as a secure emotional base, providing the necessary psychological safety to actively explore their environment and internalize a sense of meaning in life (Chen et al., 2023; Hu et al., 2023). This systemic perspective aligns with the OSI theory, positioning the family as a foundational micro-system that facilitates successful social integration within school environment (Xin et al., 2023).

### 2.4 School belonging

The concept of school belonging, as defined by Goodenow and Grady (1993), pertains to the degree to which students feel academically and emotionally accepted, supported, respected, and included by their peers and educators. A strong sense of school belonging not only enhances students’ feelings of safety and perceptions of fair treatment, but also fosters a positive perception of teachers as caring and supportive figures (Wong et al., 2022). Students who feel accepted and welcomed by teachers often have positive feelings of satisfaction and joy, while students who feel rejected, neglected, or excluded usually experience negative emotions (Lee and Huang, 2021).

### 2.5 Family functioning and meaning in life

Previous studies demonstrated that family functioning have a close relationship with meaning in life. Huang Z. et al. (2022) considers that family functioning can positively related with meaning in life among medical students. At the same time, Perceived family functioning emerges as a vital resource for fostering a sense of purpose. This strengthened sense of meaning can mitigate feelings of hopelessness and despair, thereby promoting their overall mental wellbeing (Zhou J. J. et al., 2023). This is consistent with the research of Zhang et al. (2021), who also found that family functioning is strongly related to the meaning of life. Teenagers are likely to look for purpose with family members’ assistance if the family functioning is healthier; while teenagers are less likely to appreciate the meaning of life if family functioning is weaker.

The family system is not merely a set of simple associations but rather the primary setting in which adolescents construct their sense of meaning. According to the circumplex model of family systems, high levels of cohesion and adaptability provide the structural stability required for existential growth (Olson et al., 1983). When family functioning is optimal, the environment provides emotional safety and consistency, which allows adolescents to redirect their cognitive attention away from survival concerns and toward active pursuit of life goals (Steger et al., 2006). Moreover, cohesive family groups possess numerous shared advantages, including a sense of belonging and collaborative goal setting, which directly linked to adolescents’ own sense of meaning (Goodman et al., 2019). Therefore, family functioning does not merely shape adolescents’ worldview. It also provides the essential cognitive and emotional framework that integrates diverse life experiences into a coherent and meaningful narrative. Based on this rational that the family system serves as the foundational scaffolding for existential development, the present study proposes the following hypothesis:

*H1*: Family functioning can positively affect the meaning in life.

### 2.6 Family functioning and school belonging

The Overlapping Spheres of Influence (OSI) theory provides the framework for understanding the intrinsic relationship between family functioning and school belonging. This theory emphasizes that family and school are not isolated, separate spaces but overlapping ecosystems that collaboratively shape adolescents’ developmental outcomes. From this perspective, family functioning constitutes the most fundamental source of emotional support and cognitive resources that students bring with them into the school setting.

Empirical research supports this, showing that students from families with greater cohesion and adaptability, which are the main parts of the family functioning, are more likely to experience a stronger sense of belonging at school (Xin et al., 2023; Yao et al., 2022). This suggests that a nurturing family environment promotes positive school interactions. Family functioning play a crucial role, influencing various factors like children’s mental health (Whelan et al., 2024). For example, students from families with higher educational backgrounds often feel a stronger sense of belonging at school, likely due to the increased engagement and cognitive support provided by well-educated parents (Allen et al., 2018; Law et al., 2013). Through daily positive interactions, a well-functioning family cultivates adolescents’ interpersonal skills and emotional regulation, which naturally translates into better adaptability and peer integration within the school context. Therefore, this study proposes the following hypothesis:

*H2*: Family functioning can positively affect the school belonging.

### 2.7 School belonging and meaning in life

The conceptual connection between school belonging and the meaning of life is rooted in the basic needs of human beings for belonging (Baumeister and Leary, 1995). According to the theory of self-determination, belonging is a core psychological nutrition, which can support the development of coherent and targeted self-identity (Ryan and Deci, 2017). When teenagers feel the acceptance and attention of the school community, they will experience a sense of importance, that is, they believe that their existence is meaningful to others, which is the main cognitive premise for developing a lasting sense of meaning in life (Allen et al., 2018).

Empirical research further confirms this relationship. The findings of Ramirez et al. (2021) suggest that adjustable educational factors, including the sense of school belonging, could be utilized in preventive interventions to enhance both academic achievement and the mental health among children and adolescents. Yıldırım et al. (2023) suggests that adolescents are actively searching for deeper meaning in life, and a robust sense of school belonging can foster a supportive environment that enables adolescents to cultivate meaning in life. When adolescents experience a sense of belonging, they are more inclined to participate in school in a manner that feels meaningful, ultimately enhancing their overall life satisfaction. This is in line with the research of Zhang et al. (2018), who demonstrated that a broad sense of belonging has been identified as the predictor of life meaning. Given that emerging adults dedicate significant time to school for personal growth or career development, developing a closer social bond to their education may also help them feel more purposeful in life. Therefore, this study proposes the following hypothesis:

*H3*: School belonging can positively affect the meaning in life.

### 2.8 The mediating role of school belonging

The Overlapping Spheres of Influence (OSI) framework contends that families and schools do not operate as disconnected parallel institutions, but as overlapping, mutually reinforcing systems. Resources cultivated within one domain often require reinforcement from the other to achieve their intended developmental impact (Epstein et al., 2002). This theoretical insight provides a crucial analytical framework for understanding the pathways through which family functioning fosters adolescents’ meaning in life.

Specifically, well-functioning families provide adolescents with a range of internal resources, including emotional security, basic trust in interpersonal relationships, and a coherent sense of self-worth (Bowlby, 2003). However, these internal resources alone may not be sufficient for adolescents to truly understand why they live. As Steger et al. (2006) point out, a sense of meaning in life stems not only from intrinsic value inheritance but also requires continuous social validation through interpersonal interactions. Entering early adolescence, an individual’s social focus shifts significantly to peers and the school environment (Erikson and Erikson, 1998; Ferguson et al., 2022), making the school setting the primary venue for testing and realizing the psychological resources provided by the family.

Better family functioning is conducive to providing students with more stable and effective family support. Strong family functioning helps students integrate into peer groups and build positive relationships with teachers and classmates, which can help them adapt more easily to campus life and foster a sense of school belonging. This sense of school belonging enables students to experience positive emotions, recognize the meaning of meaning and appreciate the value of a fulfilling life, which ultimately strengthen their overall meaning of life (Jiang et al., 2022). What’s more, like other cohesive groups, cohesive families enjoy the same advantages, including belonging, shared goal setting, and meaning in life. The strongest sense of cohesiveness and belonging is typically found in intimacy groups, such as families, which can affect the meaning in life at last (Goodman et al., 2019). Therefore, this study proposes the following hypothesis:

*H4*: School belonging can be a mediator in the association between family functioning and meaning in life.

**Figure 1:**
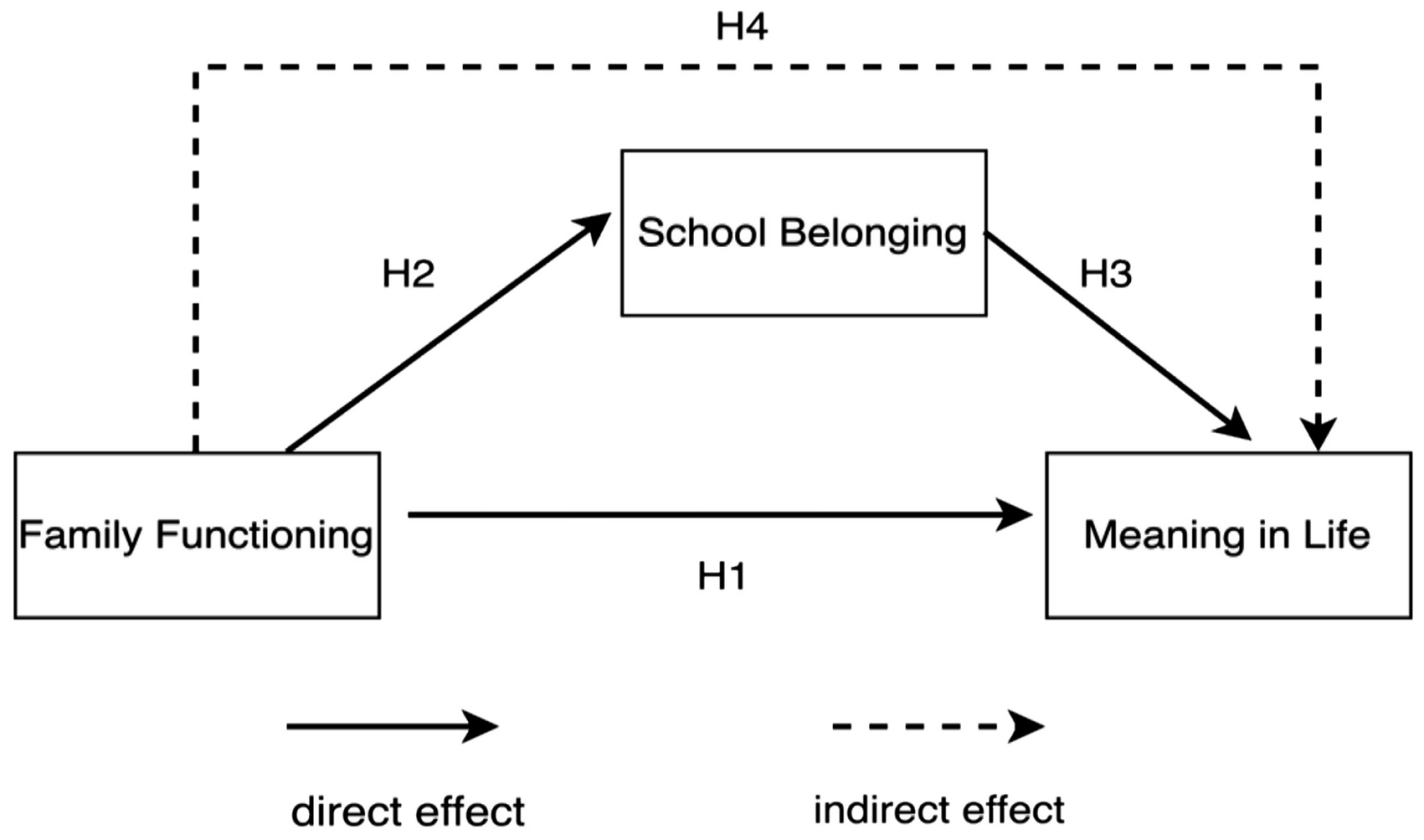
Research framework.

Figure 1 displays the research framework.

## 3 Research method

### 3.1 Instruments

#### 3.1.1 Family functioning

Family functioning was evaluated using the Family APGAR Questionnaire developed by Smilkstein (1978). This instrument is designed to assess an individual’s satisfaction with five core factors of family functioning, including adaptation, partnership, growth, affection, and resolve. The questionnaire contains five questions, and it is scored in three levels, from “hardly ever” to “almost always,” with 0–2 points, respectively. A higher score indicates better family functioning. A sample item includes “I am satisfied that I can turn to my family for help when something is troubling me.” For the present study, the Chinese version of the Family APGAR was used, which has been widely validated by scholars (Huang S. et al., 2022; Wang et al., 2025). In this research, Cronbach’s alpha is 0.823.

#### 3.1.2 School belonging

This study utilized the Psychological Sense of School Membership Scale (PSSM) compiled by Goodenow and Grady (1993) to measure school belonging, which includes 18 items. The PSSM measures perceived liking, encouragement, acceptance, inclusion and respect. The scale adopts a five-point scoring method, with 1–5 indicating from “never like this” to “always like this,” in which five items (D3, D6, D9, D12, D16) are coded in reverse. Increased scores correspond to a heightened sense of school belonging among individuals. A sample item includes “I feel like a real part of this school.” For the present study, the Chinese version of the PSSM was adopted, which has been widely validated among Chinese scholars (Yun et al., 2026; Zhang J. et al., 2024; Zhang Z. et al., 2024). In this research, Cronbach’s alpha is 0.865.

#### 3.1.3 Meaning in life

The 10 items of meaning in life questionnaire (MLQ) compiled by Steger et al. (2006) was adopted to measure meaning in life. The tool evaluates both the presence of meaning and search for meaning. The scale adopts a 7-likert scale, with scores of 1–7 from “very disagree” to “very agree,” including one item (B9) in reverse scoring. As scores increase, the degree to which an individual feels a sense of meaning in life also rises. The validated Chinese version of the MLQ was implemented. A sample item is “I understand my life’s meaning.” In this research, the Cronbach’s alpha is 0.895.

### 3.2 Power analysis

G*power software can be utilized to perform an *a priori* power analysis (Hair et al., 2014). F-test was used in the program setting, with effect size equaled to 0.15, a power of 0.80, and the significance level of 0.05. The G*power program calculated the required minimum sample size is 77. According to Comrey and Lee (2013),the participant in this study is over 77 and is thus regarded as good size.

### 3.3 Participants and procedures

This study employed a multi-stage stratified sampling approach to recruit participants. This method was specifically chosen to ensure a highly balanced representation of the adolescent population while minimizing the intra-class correlation commonly observed when surveying intact classrooms.

The sampling procedure was conducted in four explicit stages. First, 4 public middle schools in Chenzhou were selected. Second, the student population was stratified across 3 grade levels (Grades 7, 8, and 9) to ensure a representative sample of adolescence. Third, 4 classes were randomly selected from each grade level within the participating schools, resulting in a total pool of 48 classes. In the final stage, rather than sampling entire classrooms, exactly 10 students were randomly selected from each of the 48 classes to participate in the study. Through this rigorously structured process, a total of 480 questionnaires were distributed. Prior to formal hypothesis testing, strict data screening was performed. Seventy responses were excluded due to incomplete data or identifiable unengaged response patterns. Consequently, the final valid sample comprised 410 students, yielding a highly realistic effective response rate of 85.42%. This final sample size provided robust statistical power, well exceeding the recommended thresholds for conducting mediation analyses (Hair et al., 2016).

This study adhered to ethical standards with full consideration for the rights and welfare of human participants. This research was approved by the Technical Research Ethics Committee of the Affiliated Hospital of Xiangnan University with ethics approval number K2026-026-01. Prior to data collection, formal administrative approval was acquired from the relevant authorities of the participating institutions. Given that the participants were minors, informed consent was also obtained from their parents or legal guardians, ensuring they were fully aware of the study’s purpose and procedures. Each paper questionnaire began with a formal informed consent section that detailed the study’s purpose, its significance, and the strict academic use of the gathered data. Participants were required to read and explicitly select the agree option to proceed to the main body of the survey. This process guaranteed that all participation was completely voluntary and that all respondents were fully apprised of their rights and the anonymity of their data.

Table 1 displays the demographic characteristics of the final sample (*N* = 410). The sample comprises 178 males (43.4%) and 232 females (56.6%). Regarding their grades, 154 students are in grade 8. Followed by 150 students in grade 9, and 106 students in grade 7. Additionally, only 30 participants (7.3%) are the only child in their families, whereas the remaining 380 (92.7%) have one or more siblings. Meanwhile, the participants’ ages range from 12 to 16 years, with a mean age of 13.3 years (*SD* = 0.898).

**Table 1:** Demographic information (*N* = 410).

Variables	Items	Frequency	Percent (%)
Gender	Male	178	43.4
	Female	232	56.6
Grade	Grade 7	106	25.9
	Grade 8	154	37.6
	Grade 9	150	36.6
One child or more	One child	30	7.3
	More than one child	380	92.7
	Total	410	100

Variables	Mean (SD)
Age	13.3 (0.898)

### 3.4 Data analysis

This study used the Smart PLS 3.3.2 version as the statistical tool to analyze the data (Ringle et al., 2015). Model evaluation in PLS is performed by evaluating both measurement and structural models as suggested by Anderson and Gerbing (1988), and further recommended by Ramayah et al. (2018). Following a rigorous two-stage procedure, the measurement model was first evaluated to ensure data quality, specifically focusing on internal consistency reliability (via composite reliability, CR), convergent validity, which includes factor loadings and average variance extracted (AVE), and discriminant validity using the Heterotrait-Monotrait (HTMT) ratios. The cutoff values are CR > 0.7, AVE > 0.5, factor loadings>0.5, HTMT<0.85 or HTMT<0.9 (Fornell and Larcker, 1981; Ramayah et al., 2018). Upon establishing a valid measurement model, the structural model was assessed to test the collinearity issues (VIF), the significance and relevance of the relationships, *R*^2^, the effect size (*f*^2^), and the predictive relevance (*Q*^2^). According to (Kock, 2015), the cut-off value of the VIF should be 5. As suggested by (Cohen, 1988), *R*^2^ values of 0.02, 0.13, 0.26, respectively, describing weak, moderate, or substantial levels of predictive accuracy, while *f*^2^ values of 0.02, 0.15, 0.35, respectively, describing small, medium, or large effect sizes. Path coefficient value close to +1 meant a strong positive relationship and the value near −1 demonstrated a strong negative relationship, while the value close to 0 represented a week relationship (Hair et al., 2017; Ramayah et al., 2018). The statistical significance of the relationship was determined via a non-parametric bootstrapping procedure with 5,000 resamples to generate 95% Bias-Corrected and accelerated (BCa) confidence intervals, applying a one-tailed test where t-values exceeding 2.33 were considered significant at the 0.01 level.

## 4 Results

Considering that the data in this study came from self-reported questionnaires, with the same respondents serving as the only data source for both independent and dependent variables, it is critical to assess common method bias (Podsakoff et al., 2003). This study first tested the problem of common method bias by testing the full collinearity (Kock, 2015). To assess such collinearity issues, VIF was used for this evaluation. Table 2 shows that all VIF values are within 5, demonstrating that there was no problem with common method bias.

**Table 2:** Full collinearity result.

Variable	FF	MIL	SBL
VIF	1.259	1.493	1.570

### 4.1 Measurement model

The measurement model followed the steps suggested by Hair et al. (2017) and Ramayah et al. (2018), which included the assessment of validity and reliability. As displayed in Table 3, all the loadings, AVE and CR values met the threshold. This finding demonstrated that the measurements possessed both reliability and validity.

**Table 3:** Measurement model.

Constructs	Items	Loadings	CR	AVE
FF	FF1	0.756	0.835	0.585
	FF2	0.764		
	FF3	0.695		
	FF4	0.787		
	FF5	0.817		
MIL	MIL1	0.730	0.903	0.519
	MIL10	0.715		
	MIL2	0.758		
	MIL3	0.750		
	MIL4	0.754		
	MIL5	0.766		
	MIL6	0.740		
	MIL7	0.674		
	MIL8	0.762		
	MIL9	0.521		
SBL	SBL1	0.760	0.913	0.502
	SBL11	0.718		
	SBL12	0.596		
	SBL13	0.744		
	SBL14	0.704		
	SBL15	0.754		
	SBL17	0.685		
	SBL18	0.727		
	SBL2	0.657		
	SBL4	0.755		
	SBL7	0.688		
	SBL8	0.700		

SBL3, SBL5 and SBL6 were deleted because of low loadings. SBL9, SBL10 and SBL16 were deleted to achieve AVE. FF, family functioning; MIL, meaning in life; SBL, school belonging.

Furthermore, discriminant validity is evaluated using HTMT (Henseler et al., 2015). Table 4 shows the result of discriminant validity. The result showed that the HTMT values were all lower than 0.90, indicating the discriminant validity was obtained.

**Table 4:** Discriminant validity.

Variable	FF	MIL	SBL
FF			
MIL	0.397		
SBL	0.483	0.603	

### 4.2 Structural model

This research constructed three direct hypotheses among the variables. As shown in Table 5, family functioning (*β* = 0.164, *p* < 0.01) can positively affect the meaning of life among students. Thus, H1 was supported. Meanwhile, family functioning (H2, *β* = 0.424, *p* < 0.01) was significantly positively affected students’ school belonging, with an *R*^2^ of 0.179, explaining 17.9% of variances in school belonging. Moreover, the result displayed that school belonging (H3, *β* = 0.485, *p* < 0.01) was positively related with meaning in life. In addition, when testing the effect of the predictors on meaning in life, the *R*^2^ was 0.330, which meant the variables explained 33% of variances in meaning in life, showing a substantial level of predictive accuracy. Table 6 displayed that the *Q*^2^ are all more than 0, indicating that the model had sufficient predictive relevance. Furthermore, it can be observed from Table 5 that family functioning (0.219) had a medium effect in producing the *R*^2^ for school belonging, and school belonging (0.288) had a medium effect in producing the *R*^2^ for meaning in life. However, the evaluation of effect sizes demonstrated a small effect of family functioning on meaning in life (0.033).

**Table 5:** Hypotheses test.

Hypotheses	Relationships	Std *β*	*SE*	*T* values	*p* values	BCI LL	BCI UL	*f* ^2^
H1	FF- > MIL	0.164	0.046	3.603	*p* < 0.01	0.075	0.253	0.033
H2	FF- > SBL	0.424	0.041	10.422	*p* < 0.01	0.337	0.498	0.219
H3	SBL- > MIL	0.485	0.046	10.514	*p* < 0.01	0.381	0.566	0.288
H4	FF - > SBL - > MIL	0.205	0.028	7.470	*p* < 0.01	0.150	0.258	

**Table 6:** Result of *R*^2^ and *Q*^2^.

Constructs	*R* ^2^	*Q* ^2^
SBL	0.179	0.087
MIL	0.330	0.163

**Figure 2:**
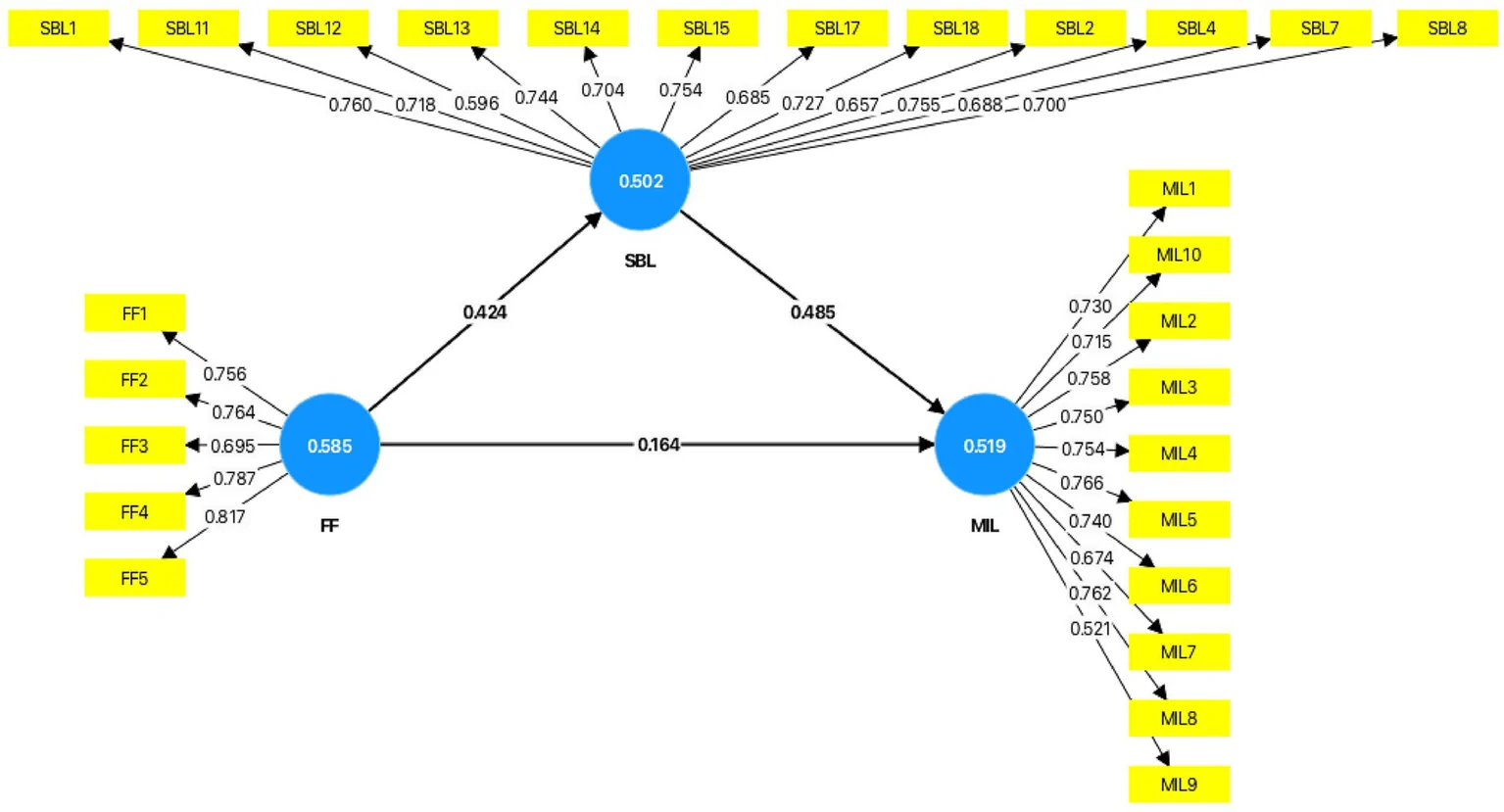
Research model.

This study conducted mediation analysis to test H4. The result indicated that family functioning (*β* = 0.205, *t* = 7.47, *p* < 0.01, 95% Boot CI Bias Corrected: LL = 0.150, UL = 0.258) can indirectly affect the meaning of life through school belonging. Therefore, H4 was supported. Table 7 presents the mediation analysis, which further specifies the mediating role of school belonging in linking family functioning to meaning in life. The total effect of family functioning on meaning in life was significant [*β* = 0.369, 95% CI (0.271, 0.447)]. After the mediator was added, the direct effect remained significant but decreased noticeably [*β* = 0.164, 95% CI (0.075, 0.253)]. The indirect effect via school belonging was *β* = 0.205 [95% CI (0.150, 0.258)], accounting for 55.6% of the total effect. This substantial proportion empirically demonstrates that the influence of family functioning on adolescents’ meaning in life is largely transmitted indirectly through school belonging. Figure 2 displays the research model.

**Table 7:** Mediation analysis of school belonging between family functioning and meaning in life.

Effect type	Std *β*	*SE*	95% Bootstrap CI [LL, UL]	Ratio to total effect (%)
Total effect	0.369	0.044	0.271, 0.447	
Direct effect	0.164	0.046	0.075, 0.253	
Indirect effect	0.205	0.028	0.150, 0.258	55.6%

## 5 Discussions and implications

### 5.1 Discussion

This study detected the influence of family functioning on middle school students’ meaning in life, with a focus on the mediating effect of school belonging in this association. Rather than viewing these relationships in isolation, the integration of effect sizes (*f*^2^) and path coefficients reveals that family functioning influences adolescents’ existential meaning primarily via an indirect, mediated route rather than a direct one. These results carry critical implications for parents, educators, and policymakers.

The findings revealed that family functioning can positively affect meaning in life among adolescents (H1). This means that the higher the degree of family functioning, the easier it is for students to feel the meaning of life. Good family functioning provides emotional support and sense of belonging to children, help individuals cope with pressures and challenges, make them feel accepted, and thus enrich their meaning in life. This is somewhat in line with the research of Zhang et al. (2021), Huang S. et al. (2022) and Zhou Z. et al. (2023), who all found that family functioning can promote the overall meaning of life. However, a closer look at the effect size reveals that the direct path from family functioning to meaning in life yields a small effect (*f*^2^ = 0.033). This indicates that while healthy family functioning provides a vital foundation, its direct contribution to adolescents’ sense of life meaning is constrained when compared to its indirect pathways. The small effect size does not diminish the importance of family functioning. It highlights that families primarily shape meaning in life not only through direct transmission of values, but also, and perhaps more importantly, by equipping adolescents with the emotional security needed to successfully engage with school environments.

It was hypothesized that family functioning could positively affect school belonging among junior school students (H2). This research confirmed that family functioning can positively affect students’ school belonging, with a medium effect size (*f*^2^ = 0.219). This is in line with the research of Xin et al. (2023) and Whelan et al. (2024). In a family with strong family functioning, students are more likely to get help and support from their parents. Parents can guide their children to deal with their emotions correctly and help them adapt to campus life actively, which can generate recognition and belonging to the school. The medium effect size indicates that family functioning is a meaningful but not overwhelming contributor to school belonging, suggesting that other factors may also play substantial roles in shaping adolescents’ sense of school integration.

The finding of H3 indicated that school belonging can positively affect the meaning of life, which aligns with the research of Ramirez et al. (2021) and Yıldırım et al. (2023). The effect was substantially larger than the direct effect of family functioning (*f*^2^ = 0.288, approaching a large effect). When students feel acceptance and support from the school community, they will directly enhance their positive experience of life, thus making it easier to perceive the meaning of life. The large effect size underscores that school belonging is a particularly potent proximal determinant of adolescents’ existential meaning during adolescence.

This study also found that school belonging plays a mediating role in family functioning and meaning in life (H4). This aligns with the research of (Jiang et al., 2022). Notably, the indirect effect (*β* = 0.205) was larger than the direct effect (*β* = 0.164), indicating that most of the family functioning’s influence on meaning in life is transmitted through school belonging. This statistical contrast highlights a specific difference between the two developmental pathways. The direct effect suggests that a healthy family environment provides a baseline, independent source of existential meaning. However, the stronger indirect effect reveals that the family’s primary developmental role is to equip students with the necessary support to adapt and develop school belonging. Consequently, this belongingness acts as the dominant mechanism where adolescents’ sense of meaning is actively constructed and validated. This finding offers strong empirical support for the OSI theory’s central proposition, that developmental outcomes are most strongly shaped at the intersection of multiple, overlapping spheres of influence.

This paper contributes to existing literature in several fundamental ways. First, in terms of understanding the mechanisms through which family and school systems jointly shape meaning in life, this study responds to calls for more integrated models of adolescent development by demonstrating that school belonging mediates the relationship between family functioning and meaning in life among Chinese adolescents. While previous research has separately established links between family functioning and meaning in life (Huang J. et al., 2022) and between school belonging and meaning in life (Kiely and Butterworth, 2013; Yıldırım et al., 2023), few studies have tested an integrated model that examines how these constructs operate together. By revealing that family influences on meaning are partially transmitted through adolescents’ experiences in the school context, this study advances theoretical understanding by showing that meaning in life is shaped by the interplay between multiple developmental systems rather than by family or school factors in isolation.

Second, the identification of school belonging as a mediator revealing that family functioning shapes meaning in part by equipping adolescents to form meaningful connections in the school environment. This finding fills a key gap in existing literature by applying the OSI theory to individual existential outcomes. Previous research has largely viewed family and school as parallel systems predicting mental health, but this study critically reveals their synergistic pathways. Meaning in life is not simply an individual trait, but rather a product of the overlapping effects of multiple environmental supports. Family functioning provides a psychological safety net for students entering the broader microsystem of school by cultivating their adaptability and emotional foundation, while a sense of belonging to school provides the direct social validation needed to translate these resources into meaning in life.

Third, this study focuses specifically on adolescents, addressing the gap in research on meaning in life during this unique developmental window. By demonstrating that family functioning and school belonging significantly predict meaning in life during early adolescence, this study shows that meaning-making processes are active and malleable during this period, when identity formation and the search for meaning are most volatile.

### 5.2 Theoretical implication

The theoretical implications of this study are reflected in the following three aspects. First, this research expands the application scope of the OSI theory. While traditional OSI theory primarily focuses on the impact of home-school partnerships on students’ academic achievement or behavioral norms, this study introduces it into the field of life education for adolescents. By exploring the collective influence of family and school systems on the profound spiritual domain, the sense of meaning in life, this study validates the universality and explanatory power of the theory within the framework of positive youth development.

Second, this study reveals the internal pathways through which multiple spheres of influence affect individual life values. By introducing school belonging as a mediating variable, this research not only confirms the direct effect of family functioning but also articulates the dynamic mechanism by which the family sphere crosses boundaries to enhance psychological connections within the school sphere, thereby elevating the sense of meaning in life. This finding deepens the logical explanation of how systemic interactions are transformed into individual psychological resources within OSI theory, providing a refined theoretical model for understanding the process of psychological construction in adolescents.

Finally, this study reinforces a holistic perspective in adolescent mental health research. The results strongly support the core hypothesis of OSI theory: that family and school are not isolated sources of influence; rather, the degree of overlap and the quality of interaction between them are critical to the acquisition of a sense of meaning. This breaks through the limitations of traditional single-environment determinism and analyzes the roots of meaning-seeking among adolescents from the perspective of systemic integration, contributing new empirical evidence in educational practice.

### 5.3 Practical implications

The findings of this study offer critical practical implications across educational, clinical, and policy domains. Importantly, family functioning should be understood as a positively valanced, malleable strength rather than merely the absence of familial dysfunction (Olson et al., 1983). Accordingly, interventions aimed at enhancing adolescents’ meaning in life should prioritize strengths-based, universal approaches that cultivate family resources proactively, complementing targeted support for families in need.

Primarily, in highly competitive academic environments, particularly within the Chinese context, schools must broaden their mission beyond mere academic achievement. Because school belonging mediates the relationship between family and life meaning, educational institutions can serve as a vital compensatory mechanism for students from less optimally functioning families. By intentionally fostering a supportive school climate and integrating life education into the curriculum, educators can provide the psychological safety needed to help adolescents navigate existential distress and build healthy value systems. Beyond these compensatory strategies, schools can also implement universal family-strengthening programs for all parents, such as workshops on parent–child communication, guided family routines, and value-oriented dialogues, that enhance family cohesion and adaptability before problems emerge, thereby reinforcing adolescents’ existential resources at the source.

Furthermore, for mental health professionals working with early adolescents experiencing apathy or existential meaninglessness, this study underscores the necessity of moving beyond individual psychopathology. Clinicians must utilize a systemic assessment approach that evaluates the overlapping spheres of a youth’s life, specifically assessing family dynamics alongside the degree of school integration. Effective interventions should actively rebuild the adolescent’s social fabric by combining family counseling with school-based collaboration, ensuring that all critical contextual factors are addressed in the therapeutic process. Effective interventions, however, are not limited to clinical counseling. Practitioners can also act as consultants and facilitators, training parents in evidence-based parenting practices, coaching teachers on how to foster classroom belonging, and brokering collaborations between families and schools. These roles extend the clinician’s impact beyond the therapy room and into the everyday contexts that shape adolescents’ existential development.

Finally, from a macro-level perspective, policymakers must view the cultivation of positive school climates and family support systems as direct investments in the existential resilience of the next generation. Rather than focusing solely on reactive crisis management, mental health funding and educational policies should prioritize preventative, social-ecological initiatives. Because early adolescence is a volatile period for meaning-making, policy frameworks must promote the seamless integration of family, school, and community resources to collectively safeguard youth wellbeing. Specifically, policymakers could consider incorporating routine family functioning screenings (e.g., the Family APGAR) into school health assessments, not as a diagnostic tool for labelling at-risk families, but as a proactive mechanism for identifying families that could benefit from low-threshold, educational strengthening programs. Such an approach reframes family functioning as a continuously developable advantage rather than a static deficit.

This study has several limitations that should be recognized. Firstly, this study is cross-sectional research, restricting the ability to infer causal relationships. While the mediation model was theoretically grounded, longitudinal research is needed to establish temporal precedence and examine developmental trajectories across the junior high school years. Second, the use of only self-report assessments brings up issues related to common method variance, and future studies should include multiple sources of information and different measurement approaches. Third, the sample was drawn from a single city in China. Future research should examine these relationships in more diverse samples across different regions and cultural contexts. Fourth, the study examined only one mediator, and future research should investigate additional mechanisms such as self-esteem, psychological resilience, and identity formation that may also explain how family functioning influences meaning in life during early adolescence.

Despite these limitations, this study advances understanding of how family functioning and school belonging jointly shape meaning in life among Chinese adolescents, addressing critical gaps in knowledge about the internal mechanisms linking family dynamics to existential outcomes, the mediating role of school belonging, and the unique developmental context of early adolescence.

## 6 Conclusion

This study explored the influence of family functioning on adolescents’ meaning in life. Moreover, it also examines the mediating influence of school belonging on the link between family functioning and sense of meaning in life. The findings reveal that both family functioning and school belonging can significantly influence students’ meaning in life. Moreover, it also reveals that school belonging can mediate the relationship between family functioning and sense of meaning in life.

## Data Availability

The original contributions presented in the study are included in the article/supplementary material, further inquiries can be directed to the corresponding author.

## Appendix

**Table A1:** Research questionnaire.

Variables	Items
Meaning in life	I understand my life’s meaning.
	I am looking for something that makes my life feel meaningful.
	I am always looking to find my life’s purpose.
	My life has a clear sense of purpose.
	I have a good sense of what makes my life meaningful.
	I have discovered a satisfying life purpose.
	I am always searching for something that makes my life feel significant.
	I am seeking a purpose or mission for my life.
	My life has no clear purpose.
	I am searching for meaning in my life.
Family functioning	I am satisfied that I can turn to my family for help when something is troubling me.
	I am satisfied with the way my family talks over things with me and shares problems with me.
	I am satisfied that my family accepts and supports my wishes to take on new activities or directions.
	I am satisfied with the way my family expresses affection, and responds to my emotions, such as anger, sorrow, or love.
	I am satisfied with the way my family and I share time together.
School belonging	I feel like a part of my school.
	People at my school notice when I am good at something.
	It is hard for people like me to be accepted at my school.
	Other students in my school take my opinions seriously.
	Most teachers at my school are interested in me.
	Sometimes I feel as if I do not belong in my school.
	There is at least one teacher or adult I can talk to in my school if I have a problem.
	People at my school are friendly to me.
	Teachers here are not interested in people like me.
	I am included in lots of activities at my school.
	I am treated with as much respect as other students in my school.
	I feel very different from most other students at my school.
	I can really be myself at my school.
	Teachers at my school respect me.
	People at my school know that I can do good work.
	I wish I were in a different school.
	I feel proud to belong to my school.
	Other students at my school like me the way that I am.
